# P05.40. Traditional medicine and primary health care: leading examples from around the World

**DOI:** 10.1186/1472-6882-12-S1-P400

**Published:** 2012-06-12

**Authors:** D Hollenberg, M Torri

**Affiliations:** 1 Royal College of Physicians and Surgeons of Canada, Ottawa, Canada; 2 University of New Brunswick, Department of Sociology, Fredericton, Canada

## Purpose

This paper will draw on recent ethnographic fieldwork to present and discuss leading examples of how traditional/indigenous medicine (TM) is used as primary healthcare (PHC) – or first point of contact – for diverse international communities. For the past 30 years, TM has been identified by policy-makers such as the WHO as essential for successful PHC, yet international development programs have yet to fully engage with TM projects “on the ground”.

## Methods

Data in this paper will draw on extensive ethnographic fieldwork within the identified countries under discussion.

## Results

Two different and recent case studies will be presented to highlight the discussion: (1) Home Herbal Gardens (HHGs) in Bangalore, South India; and (2) TM practices in South East Asia (Indonesia). The data presented will focus on TM practices that enhance PHC along with social capital and community health. Using visual footage of TM practices, the presentation will conclude by demonstrating the vital importance of TM for PHC and community health worldwide.

## Conclusion

Drawing on the presentation and the data under discussion, an arguement for validating and extending the role of TM in PHC will be made.

